# Ellipsoidal Abstract and Illustrative Representations of Molecular Surfaces

**DOI:** 10.3390/ijms20205158

**Published:** 2019-10-17

**Authors:** Meng Liang, Yuhang Fu, Ruibo Gao, Qiaoqiao Wang, Junlan Nie

**Affiliations:** School of Information Science and Engineering, Yanshan University, Qinhuangdao 066004, Hebei, Chinafuyuhang_fly@163.com (Y.F.); grb664425@126.com (R.G.); wangqiaoqiao92@163.com (Q.W.)

**Keywords:** ellipsoidal abstract, level of detail, biological structures, molecular visualization

## Abstract

Molecular visualization is often challenged with rendering of large molecular structures in real time. The key to LOD (level-of-detail), a classical technology, lies in designing a series of hierarchical abstractions of protein. In the paper, we improved the smoothness of transition for these abstractions by constructing a complete binary tree of a protein. In order to reduce the degree of expansion of the geometric model corresponding to the high level of abstraction, we introduced minimum ellipsoidal enveloping and some post-processing techniques. At the same time, a simple, ellipsoid drawing method based on graphics processing unit (GPU) is used that can guarantee that the drawing speed is not lower than the existing sphere-drawing method. Finally, we evaluated the rendering performance and effect on series of molecules with different scales. The post-processing techniques applied, diffuse shading and contours, further conceal the expansion problem and highlight the surface details.

## 1. Introduction

Exploring the interaction between biomolecules is one of the aim of molecular visualization. Some of these biomolecules are rather large entities and are, therefore, referred to as macromolecules such as protein whose building blocks are amino acids. Since in the field of molecular visualization, the scale of protein to be explored become very large (several million to billion of atoms), a current challenge is to improve the efficiency of the interactive visualization of large proteins.

Molecules can be visualized with various degrees of structural abstraction like space fill, balls-and-sticks, licorice, backbone and ribbon [1]. Different structural abstraction stages have different rendering speed. When molecular surface is mentioned, it suggests that the structural abstraction of molecule is space fill. The three common geometric representations employed for visualizing molecular surface are solvent-excluded surface (SES), gaussian kernels and van der Waals spheres (vdW). The information contained in these three surface reduces in turn, while the performance of them increases in turn. Taking advantage of it, Parulek [2] utilizes these three geometric representations according to the distance from the viewer to form seamless visual abstraction of molecular surfaces. To improve the rendering performance of macromolecules, different levels of abstraction use models with different complexity is a typical idea of lod. In molecular visualization, a bigger geometric sphere called bounding sphere is usually used to represent a set of atom sphere. These bounding spheres constitute a lower level abstraction. But bounding sphere is not the most stringent bounding box, we choose the minimum-volume enclosing ellipsoids (MVEE) as its replacement to reduce the volume expansion.

In the field of molecular visualization, there are many molecular visual representations and interactive rendering techniques [3]. Our main focus is hierarchical geometry abstraction of macromolecule as well as shading technology. Our approach builds on several aspects of previous work on molecular visualization, in particular with respect to surface representations, visual rendering, and methods for interactive rendering especially LOD approaches. We have divided the related work into three sections accordingly.

### 1.1. Molecular Surface Representations

There are several molecular surface representations. Lee and Richards [4] first described the van der Waals spheres in 1971. That was the starting point of the invention of further types of molecular surfaces representations. The solvent accessible surface (SAS) [5], one of the first extensions of the vdW surface, shows all regions of a molecule that can be accessed by a solvent molecule which is usually approximated by a probe. SAS is defined by the centre of a spherical probe while rolling over the vdW surface. In 1978 Greer and Bush [6] gave an definition of solvent-excluded surface (SES) that also be regarded as the topological boundary of the union of all possible probe spheres that roll over the vdW surface while not intersecting with any atom of the molecule. In contrast to SAS, the SES uses the outer shell of the probe that rolls over the atoms instead of taking the center of the probe. Blinn [7] achieves gaussian Surfaces, an approximation of the molecular surface, by the use of a gaussian convolution kernel. There are several other kernels can be used as alternative kernel functions [8]. Isosurface obtained by such a kernel-based models can approximate SES with a linear time complexity. In contrast to the kernel-based approaches, Parulek [9] proposed another implicit model with linear complexity that resembles the SES more closely. Recently, Lindow et al. [10] gave a generalized molecule surface of the SES called ligand excluded surface (LES). In contrast to the SES, the LES uses the full geometry and dynamics of the ligand’s vdW surfaces instead of a approximated sphere. Apart from molecular surface models mentioned above, several molecular surface abstractions models have been established. To reduce the complexity and computation time, this approachs are aim at showing the general shape of a molecule instead of individual atoms by the use of one or more tight-fitting bounding spheres that contain the individual atoms [10,11,12].

### 1.2. Visual Rendering

The effect of molecular visualization often features a high visual complexity. Several shading techniques and methods offering various depth cues are necessary to enhance the image quality and the perception of molecular shape and depth. The Toon/Cel shading by David Goodsell [13] is often used to produce artistic or non-photorealistic renderings. This illustration approach combining cel-shading with silhouettes to enhance the perception of the overall shape of molecule has also been recently adopted by Falk et al. [14]. Similarly, Parulek [2,15] uses different shading with or without contours to form a several kinds of visual effect that inspires the creation of the renderings shown in this paper. Depth-dependent silhouettes [16] can distinct object boundaries in a post-processing step by detecting discontinuities in depth. The depth darkening approach [17] uses simple fogging and depth-dependent desaturation as additional depth cues. Halos proposed by Tarini et al. [18] extends the object boundaries by featuring the same depth as the object.

### 1.3. Interactive Rendering

Many approaches has been studied for improving the overall rendering performance of molecular data. In addition to the molecular surface abstract simplification [19], reducing the number of atoms to rendering is also a kind of effective method. For example, Sharma et al. [20] exploites octree and view-frustum to render billions of atoms interactively. Occlusion culling is often used to simplify data [21]. Lampe et al. [22] reduces the transmission data for visualization of large protein assemblies. Only orientation information of residue is sent to the GPU, then the atom positions of the residue can be reconstructed on-the-fly. Similarly to it, taking full advantage of the dynamic tessellation capabilities of modern graphics cards, Muzic et al. [23] improves the rendering performance by only sending the center of the molecules to GPU, while the reconstruction of the atoms is performed in shaders.

The level-of-detail (LOD) technique has a long history in molecular visualization. For instance, Lee et al. [24] uses LOD technique to visualize large-scale molecular models based on a bounding tree. Besides, some techniques for rendering molecular data use surface simplification methods to generate different LODs [25]. In contrast to vdW and SAS, the SES has more information for analysing molecular interactions and studying cavities but is harder to compute. Based on this, Parulek [2] utilizes this three surfaces as different level of detail to simplify surface. In 2014, Parulek [15] extra introduces the hierarchical data representation which further provides users with fine-grained control over the different representational dimensions and enables them to flexibly and seamlessly adjust the level of abstraction during interactive visualization. Falk et al. [14] extended Lindow’s [26] technique by a hierarchical LOD to accelerate the rendering: if the projection of a grid cell is smaller than a pixel, it is not necessary to perform a ray casting with the spheres in a cell. With the increasing distance to the camera, Muzic et al. [23] discard more insignificance atoms and scale the radius of the remaining atoms accordingly. Certainly his methods can essentially create LOD.

In the field of molecular visualization, we find it is a common practice to use simplified models to improve efficiency. In order to reduce the expansion problem caused by it, we propose a LOD algorithm that use not only a special clustering algorithm but also a new enclosing geometry. Of course, some visual effect technologies are need to enhance the perception of molecular shape and depth.

## 2. Result Analysis And Limitations

To illustrate the performance we give rendering frame-rates on a 2.4 GHz i7 with 16 GB memory and a Nvidia Geforce GTX 780Ti (4 GB memory card) run under 64bit windows for a range of molecule sizes. We use Megamol [27] as our rendering platform. The frame rates are given for the molecules rendered at approximately 1920 × 1080 pixels for the all measurements.

**Figure 1:**
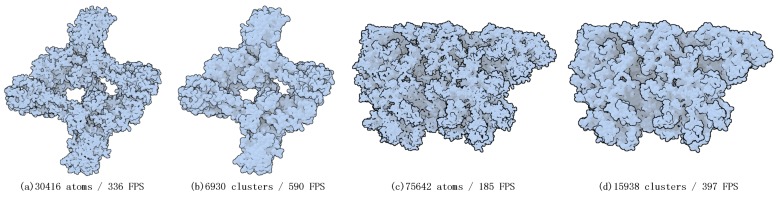
Two molecular examples, a transferase protein and a viral protein, demonstrating utilization of our ellipsoidal abstract representations (**b**,**d**). In the presented examples we achieved 1× to 2× speed-up as compared to the full vdw representation (**a**,**c**).

In Figure 1, each amino acid of two proteins was processed into a cluster and represented by an ellipsoid. The reason why we did this experiment is that in the field of molecular visualization, the amino acid composition of proteins is worthy of concern [28]. Figure 1 shows how much efficiency can be improved if only the amino acids of the protein are drawn.

**Figure 2:**
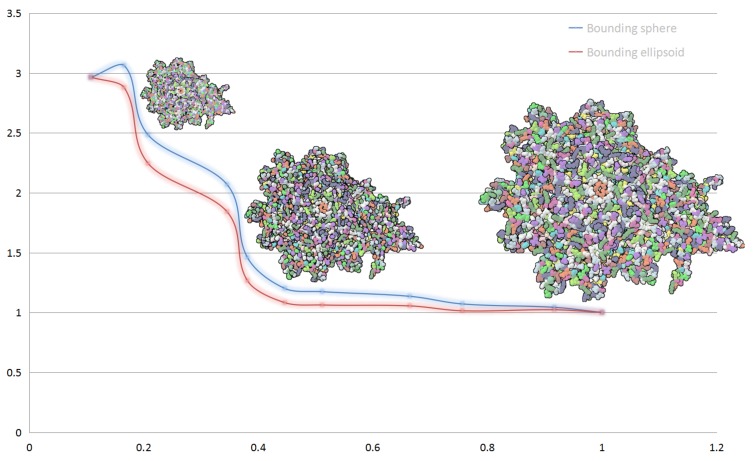
Performance evaluation of hierarchical representation. The horizontal axis of chart represents the ratio of the cluster render list size to the full atom count. The vertical axis represents the speed-up of rendering performance of hierarchical representation to full vdW representation.

In Figure 2, we show the performance of our hierarchical abstract and compare the performances when applying different bounding box for a same display list. Blue line shows the frame rate change of bounding sphere representation and red line shows bounding ellipsoid representation. The performance is slightly lower when using ellipsoid model for clusters than using sphere model. At the beginning of simplifying, the performance gap is little, with the increase of the distance between camera and protein, the rendering performance of molecular models get even up to 3× compared to full vdW representation. The rendering performance of full vdW representation is about 1000 fps.

**Figure 3:**
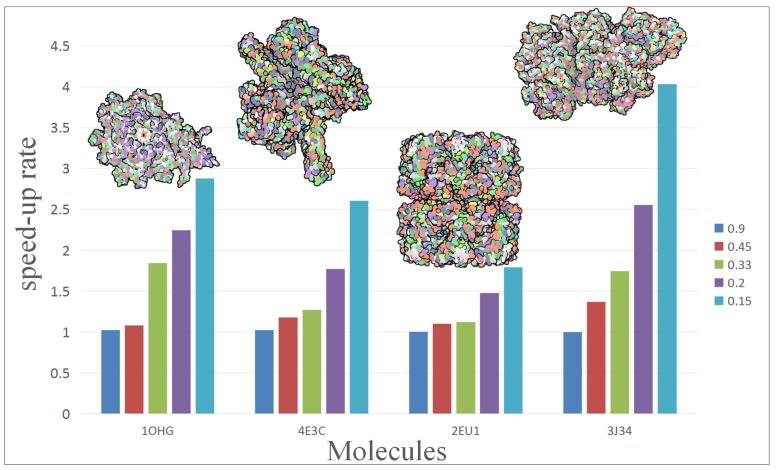
Show the performance improvement of different scale of molecules with different degree of simplification. The molecule contains from left to right: 15,057, 30,416, 53,970 and 75,642 atoms.

To further analyze and compare the performance of our approach, we rendered a test scene with different scale of molecules. Figure 3 shows the performance change of four molecules with five simplification. The vertical axis of chart represents the speed-up of hierarchical representation to vdW representation. The molecule contains from left to right: 15,057, 30,416, 53,970 and 75,642 atoms. In the present experiment, different molecules get a different acceleration effect: 2EU1 just achieved 1.8× speed-up while 3J34 can even get up to 4×.

**Figure 4:**
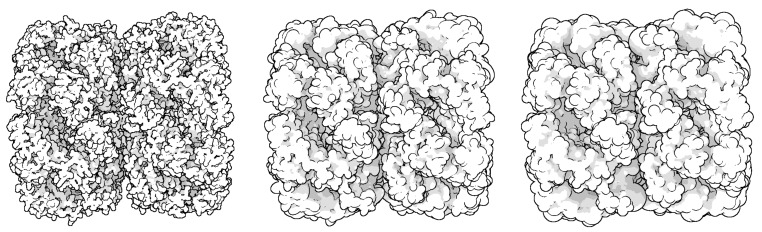
Comparison of three representation of the molecule (ID: 2EU1) performed using the vdW (**left**), bounding ellipsoid (**middle**) and the bounding sphere (**right**).

**Figure 5:**
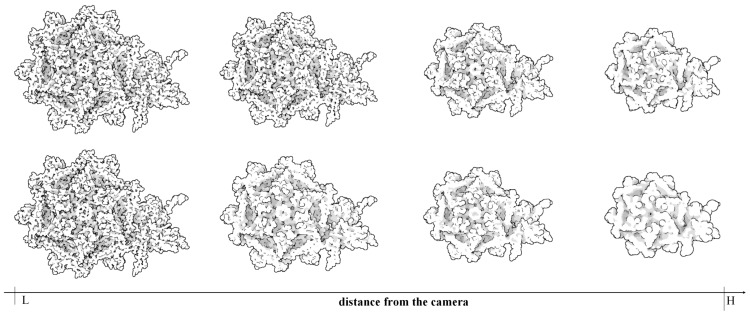
Comparison of zooming out towards the molecule (ID:1OHG) performed using the bounding ellipsoid (**top**) and the bounding sphere (**bottom**).

Through LOD technology we are able to boost the rendering performance of molecule, and meanwhile, keep the more detail of vdW representation for the closest parts of the molecule from the camera. Additionally, when applying the ellipsoidal representation, we get tight overall shape of molecule compared to the sphere representation. We compared the three representation of a molecule in Figure 4. The ellipsoidal representation has more sharp contours, and when using screen space ambient occlusion (SSAO) technology, its concave part has a higher depth than the bounding sphere representation. The top portion of Figure 5 shows the hierarchical representation of the bounding ellipsoid and the bottom portion shows the bounding sphere with silhouettes and SSAO. We compared the visual representations in four columns with progressively increasing simplification ratios from left to right. Each column has the same display list. We adjusted the parameters and camera to place the molecule in a visually symmetrical structure.

We set up a scene containing $4.0\times 10^{4}$ instances of conjugal transfer protein trwb (PDB ID: 1GKI, 20150 atoms). This gives us an effective number of approximate one billion of atoms for the data set. We only use LOD selection and frustum culling, no considering other acceleration approach. We observe that the rendering of the scene achieves an average 7.0 frames/second when viewpoint zooming. Combining with other GPU acceleration technique, such as deferred shading and depth culling [14], we believe that our method will archive better performance.

At last, we have invited some users to do a small amount of testing, which proved that our method had a certain effect on the interaction. Simple interaction operation and enough frame rate ensure the fluency when user browse the scene.

## 3. Discussion

In the presented examples we achieved up to 2×–4× speed-up as compared to the vdW representation only. The reason why this method improves performance slowly at the beginning is that for ray casting method, rendering a bigger bounding model including only a few atoms may need more rays than directly rendering those atoms. With the further simplification of the molecule, more and more atoms merge into a cluster and corresponding rays do not increase a lot, bringing the increase of frame rate.

At the initial stage of simplification, ellipsoidal representation can represent more surface detail as shown in the second column of Figure 5. When a molecule is simplified to a high degree, its ellipsoidal representation has a tighter overall shape.

The utilization of LOD and ellipsoid mode still bring two major limitations. The first one is the actual surface precision when using the full atom count compared to exploiting the cluster hierarchy and using the sphere model compared to exploiting the ellipsoid mode.Ellipsoid does not conform to the visual habit of people. We just only use shading technology to hide the most of the surface dissimilarities. The second limitation is the requirement of bounding volume computing. This has to be done for each new structure modification repetitively. In fact, if we regard the conformation of the same kind of residue as only one, we can obtain the MVEEs of Residue-Interior level and Residue level clusters easily by utilizing the repetitive nature of residues.

## 4. Methods

**Figure 6:**
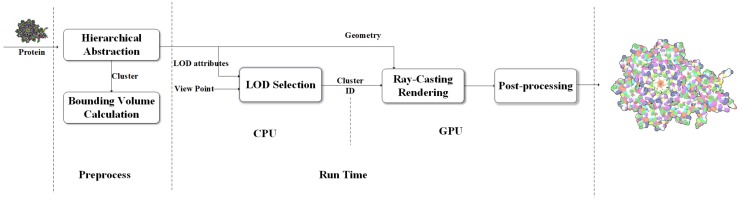
An illustration of the level of detail (LOD) rendering pipeline.Real-time computing mainly occurs in central processing unit (CPU) and graphics processing unit (GPU). At first, we get the cluster tree of molecule and compute the ellipsoids bounding box for each node of tree. The formation of the cluster display list is determined by Equation (2). Clusters are represented as spheres or ellipsoids that are rendered through glyph-based ray-casting algorithm with diffuse shading. In the end, we compute contours and screen space ambient occlusion.

Motivated by the need for the interactive visualization of large molecule, we designed a coarse-surface rendering framework which provides continuous transitions and more tightness overall shape from the view distance as shown in Figure 6.

Data processing continues until the calculatation of MVEE of each tree node have been done. The hierarchical tree and the MVEE are prepared for the last two steps where the LOD selection runs on the central processing unit (CPU) and the real-time ellipsoid rendering and visual effect implement on the GPU. In contrast to the work done by Parulek et al. [15], the main purpose of our pre-processing stage is to get a complete binary tree for keeping more hierarchical levels. Obviously, a protein with n atoms is accompanied with a hierarchical tree contains 2n-1 nodes. In order to form a series of compact abstract representation of the protein, each formed cluster is represented by a ellipsoid instead of a sphere. In the LOD selection stages, we choose clusters with different levels of detail by using a top-down recursion approach according to the distance to camera. If current level is not enough to have a high degree of conservation with respect to the outline of the molecule, we test the next level. After that, the geometry information of sphere model for atom and ellipsoid model for cluster are loaded into the GPU for interactive GPU-based ray-casting [27,29] rendering respectively. There are some advanced post-processing rendering techniques, i.e., deferred shading and screen-space ambient occlusion, can be used to reduce the illumination computational and enhance the depth perception. For better perception of shape, depth- dependent silhouettes and constant shading are displayed for surfaces to have a flat appearance.

### 4.1. Molecular Hierarchical Tree

**Figure 7:**
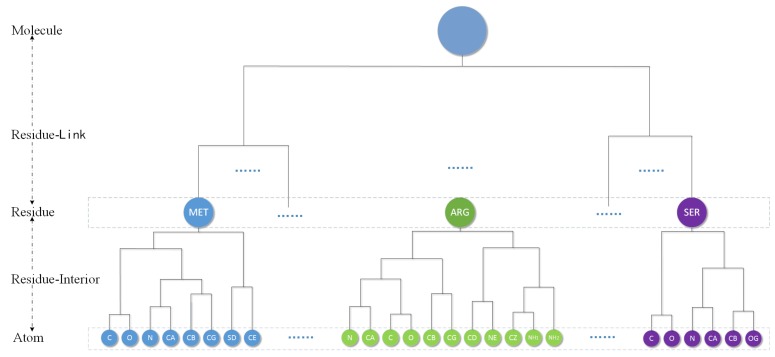
The whole cluster tree of a protein molecule from AFHC.

Considering the respect of the biological particularity of atoms, we use an AFHC (adjusted fast hierarchical clustering) method by Guo [30] to obtain the hierarchical cluster tree of a molecular. They first propose introducing a restrictive rule in the clustering process. The rule can be described as follows: the atoms in the same residue merge with each other first. It means that for the hierarchical clustering of a protein, the aggregation process occurs first between atoms/clusters in the same residue until the whole residue becomes one cluster, then the aggregation process between residues just begins. This process finally only remains a single cluster called Molecule level. A volume-based distance metric (VDM) is designed by them for spherical clustering. They compare eight common linkages and find that, except for a “single” linkage, other linkages have similar error when used with VDM. So we just choose “average” as the linkage in this paper. By using a location-based clustering and VDM, a protein molecules will produce a corresponding complete binary hierarchical cluster tree as shown in the Figure 7.

The cluster tree contains five kinds of node level: Atom, Residue-Interior, Residue, Residue-Link and Molecule. The leaves of tree are atom and every residue can be represent by one Residue level cluster. Each cluster will therefore have a single parent, and have two children. The following is a bottom-up detailed description of the hierarchy.

- ***Atom level:*** Each element is a single atom sphere. Residue-Interior levels and residue level are clustered from the atom level.
- ***Residue-Interior levels:*** Each element is a cluster of some atoms belong to only one residue. Therefore, each element contains one or several atoms. In general, they are multiple levels.
- ***Residue level:*** Residue level regards one residue as an element. This level will denote the protein primary structure. Residue-link levels are clustered from the residue level.
- ***Residue-Link levels:*** Each element is abstracted from the residues of the molecule. Therefore, each element has one or several residues and belongs to only one molecule. In general, they are also multiple levels.
- ***Molecule level:*** The highest level. The whole molecule is treated as only one element.

There are two main reasons for using residue level. First, retaining the residue sequence is helpful to understanding biology. All proteins consist of one or more linear chains of amino acids. The interconnected backbones of all amino acids are forming the basic chain. Second, there are only 20 kinds of standard amino acids, and the same kind of residue can be used for the same cluster subtree. We can obtain Residue-Interior levels easily by utilizing the repetitive nature of residues.

### 4.2. Bounding Volume Calculation

So far, cluster is handled as a bounding sphere generally. It doesn’t mean that it is not meaningless to think of an atom as an ellipsoid model [31]. Sometimes, the overall shape of the protein and the via holes can be clearly preserved. But the higher degree of the simplification is, the more obvious inflation phenomena is. The main reason is that the high level clusters involve more atoms and their geometric model is using the model of lower clusters as input, the volume of the cluster sphere becomes larger and the inflation error accumulates in clustering process. For the lower level of detail, it will lead to an visible inflation of the overall shape, resulting in an undesirable abstraction even the inflation of the overall shape will hide the via hole.

To minimize this inflation error, the VDM is used in this paper to narrow inflation from aspect of clustering instead of the common distance metrics, i.e., Euclidian distance. It changes the order of clustering elements and makes the volume of new cluster smallest. The result does well but is not enough, so we consider a new bounding volume. Compared with bounding sphere, ellipsoid, one of the common symmetrical geometry, is more efficient, flexible and compact so that the representation of cluster is fit the surface of protein molecule effectively.

**Figure 8:**
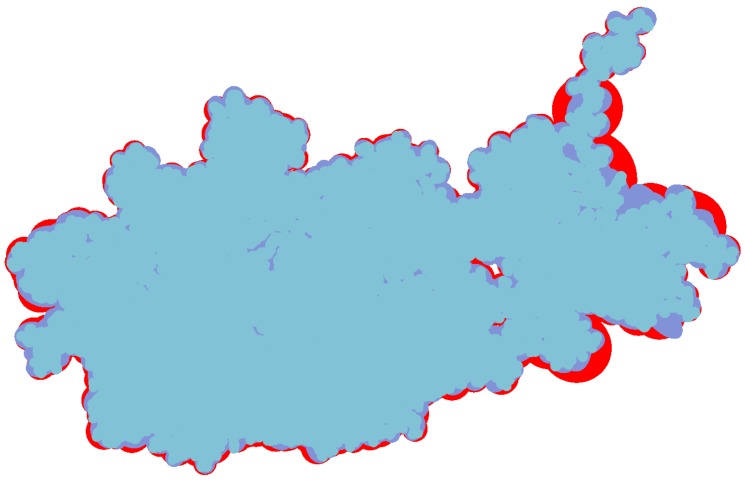
Overlap and comparison of three representations with constant shading. The bottom layer (coloured red) is bounding sphere model, the middle layer (coloured purple) is bounding ellipsoid model and the upper layer (coloured blue) is vdW surface.

The inflation caused by the increased volume of bounding box not only means the expansion of outline but also the surface inflation. So in this two aspects we discuss the advantage of bounding ellipsoid who has littler volume than other bounding box. At first we compare the constant shading surface of a protein (ID: 1OHG) with three different geometric representations. Figure 8 shows their constant shading and overlay them one by one. The bottom layer is the bounding sphere model colored red, the middle layer is the bounding ellipsoid model colored purple and the front layer is vdW surface colored blue. By adjusting the camera, the left of protein has a low simplification where the outlines of three representations are roughly the same. The right of protein has a high degree of simplification, its overall shape and via holes are more easy to be closed by bounding sphere model. Moreover, the red area expands more distinct at its bulged branch.

We used the method propose by P. Kumar [32] to compute the MVEE for each cluster except the leaves in hierarchical cluster tree which recorded the whole hierarchy of a protein. Each formed cluster is represented by an ellipsoid with a cluster centre c and three orthogonal axis, which bounds all the atoms within it. The three orthogonal axis have respective axial direction and length. In order to reduce the accumulation of inflation error, the MVEE of cluster in the Residue-Interior and Residue level of the molecule is computed by sampling points data on all the atoms within the cluster. Above the Residue level, the MVEE of next level of detail is created using the points data on the MVEE from the previous level as input.

### 4.3. Rendering

#### 4.3.1. Lod Selection

We traversed the cluster hierarchy in top–down manner to retrieve all the clusters/atoms and decide which to be used for the molecular representation and visualization. Starting from the Molecule level node of cluster tree, we evaluate whether a cluster C meets the function:

(1) $$G\left(C,D\right)\equiv D\leq \frac{dis-inival}{interval}\;$$

where *D* is the longest path between cluster *C* and its child leaves on the cluster tree, *dis* is the distance between camera and the centre of cluster *C*, preset value inival represents the distance to the camera where the protein begins to simplify and interval stands for the extent of every field. When a cluster meets the criteria or is a atom node, we add it to the display list and stop traverse this branch of cluster tree. If not, we recursively evaluate its two child nodes. After the cluster tree traversal, all the nodes that fulfill Equation (1) are added to the list. The change of camera position or parameters will call a new hierarchy traversal and send the updated display list to GPU. When the camera is farther away from the molecule, the display list becomes more reduced and the molecular representation is more abstracted than previous.

Generally some molecules always have a similar symmetrical structure. To ensure these parts can have a same representation, we introduce the restricted rule for hierarchical clustering and traverse on the whole cluster tree. Because the restricted rule in the hierarchical clustering can keep residues together. The distance-based LOD selection provides a ability to flexibly adjust the field depth and place the symmetrical structures in the same field, resulting in the symmetrical structures have the same representation.

#### 4.3.2. Ellipsoid Rendering

There are many ways to draw ellipsoids [33,34]. We use a GPU-based method that are easy to understand and calculate. An arbitrarily oriented ellipsoid, centered at c, having an orthonormal basis $v_{1}$, $v_{2}$ and $v_{3}$ with respective radius $\lambda_{i}$ as illustrated in Figure 6 can be defined as:

(2) $${\left(x-c\right)}^{T}A\left(x-c\right)=1$$

where *A* is a positive definite matrix and x, c are vectors. The eigenvectors of *A* define the principal axes of the ellipsoid $v_{i}$ and the eigenvalues of *A* are the reciprocals of the squares of the semi-axes: $\lambda_{1}^{-2}$, $\lambda_{2}^{-2}$ and $\lambda_{3}^{-2}$.

Instead of having meshes of spheres/ellipsoid, we use implicit surface rendered in the fragment shader. When rendering implicit surface, the important factors that affect the rendering time is the expensive ray-intersection calculation occur in a lot of fragments. So we place the computing operation in the vertex shader as far as possible in order to reduce the workload of the fragment shader, meanwhile use the agent geometry for fast ray casting.

**Figure 9:**
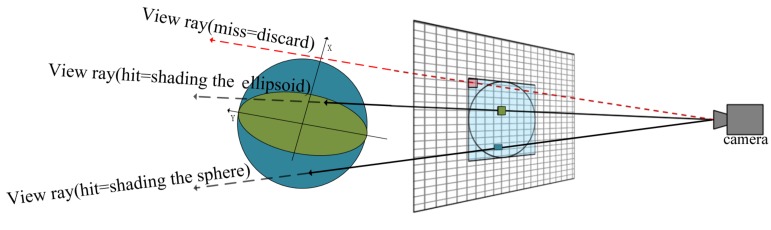
Glyph ray casting rendering of sphere and ellipsoid based on graphics processing unit (GPU)

The design for computing ray-ellipsoid intersection in this paper is illustrated in Figure 9. Due to that any ellipsoid can be generated from a sphere by stretching the sphere by the $\lambda_{i}$ in the major directions $v_{i}$. The rendering of ellipsoid can be derive easily from current GPU-based glyph ray casting rendering of sphere. In the vertex shader stage, we use the smallest agent geometry of a concentric sphere whose radius equal the longest axis of ellipsoid as the agent geometry of the ellipsoid directly. Since the sphere contains the ellipsoid, the ray through the ellipsoid must pass through the sphere as well.

In the fragment shader stage, the computation of the ray-ellipsoid intersection by using the generalized form of ellipsoid equations directly is very complex. We transform the given ray from the view location to a pixel and ellipsoid to the glyph space whose origin point is the centre of ellipsoid by rotation matrix $V=(v_{1},v_{2},v_{3})$ where the $v_{i}$ are entered as columns. Then we can solve the ray-ellipsoid intersection problem easily because the ellipsoid is now described as $\frac{x^{2}}{a^{2}}+\frac{y^{2}}{b^{2}}+\frac{z^{2}}{c^{2}}=1$. The result is used to compute the surface normal and surface location at the pixel, which is necessary for the illumination computation and the depth correction.

**Figure 10:**
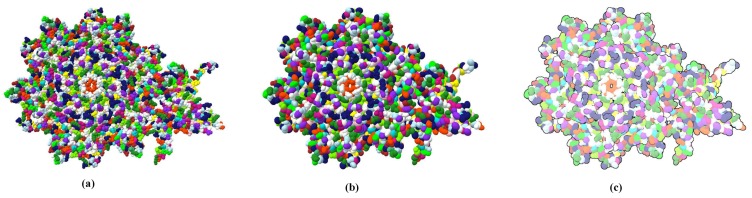
A comparison between the original van der Waals surface (**a**) and the hierarchical ellipsoid surface (**b**) of a protein (ID: 1OHG). The overall shape of the protein and the via hole are clearly preserved. The abstract surface (**c**) with diffuse shading and contours hides surface error caused by inflation.

We use the perceptual principles of object constancy to depict protein structures that are too far away to recognize the details. The details in the structural part that is closest to the camera need more prominent, while the visual prominence of overall structure of farther parts is more needed than individual details. It is important to convey the overall structure of the molecule and preserve the large-scale features. Goodsell’s novel approach is chosen to enhance the overall shape of the molecule, additionally, to increases the overall visual effect by utilizing a diffuse shading and depth-dependent silhouettes in post-processing stage in Figure 10.

## 5. Conclusions and Future Work

In this paper, we formed a hierarchical abstract representations of large molecule by utilizing the level-of-detail to reduce the amount of displayed primitives, and at the same, using the MVEE and volume-based distance metric in clustering to keep the visual appearance more similar to the original data. It can keep more surface detail and further reduce the inflation caused by bounding volume. Moreover, with a residue-considered clustering algorithms and a recurrent traversal on the whole cluster tree, we can implemente distance-dependent LOD selection and make the symmetrical structures of molecule have a same representation. We provide a link, https://github.com/angelaifox/Megamol/, to installation guides for the Megamol tool and some guidance on how to use my plug-in.

As a result of the limitations of our current work, in future work, we intend to research a smooth transition between different levels.

## References

[1] Zwan M.V.D., Lueks W., Bekker H., Isenberg T. (2011). Illustrative molecular visualization with continuous abstraction. Comput. Graph. Forum.

[2] Parulek J., Ropinski T., Viola I., Parulek J., Ropinski T., Viola I. Seamless Visual Abstraction of Molecular Surfaces. Proceedings of the 29th Spring Conference on Computer Graphics.

[3] Kozlíková B., Krone M., Falk M., Lindow N., Baaden M., Baum D. (2016). Visualization of biomolecular structures: State of the art revisited. Comput. Graph. Forum.

[4] Lee B., Richards F.M. (1971). The interpretation of protein structures: Estimation of static accessibility. J. Mol. Biol..

[5] Richards F.M. (1977). Areas, volumes, packing, and protein structure. Annu. Rev. Biophys. Bioeng..

[6] Greer J., Bush B.L. (1978). Macromolecular shape and surface maps by solvent exclusion. Proc. Natl. Acad. Sci. USA.

[7] Blinn J. (1982). A Generalization of Algebraic Surface Drawing. Conf. Comput. Graph. Interact. Tech..

[8] Sherstyuk A. (1999). Kernel functions in convolution surfaces: A comparative analysis. Vis. Comput..

[9] Parulek J., Brambilla A. (1999). Fast blending scheme for molecular surface representation. IEEE Trans. Vis. Comput. Graph..

[10] Lindow N., Baum D., Hege H.C. (2014). Ligand excluded surface: A new type of molecular surface. IEEE Trans. Vis. Comput. Graph..

[11] Tozzini V. (2005). Coarse-grained models for proteins. Curr. Opin. Struct. Biol..

[12] Clementi C. (2008). Coarse-grained models of protein folding: Toy models or predictive tools?. Curr. Opin. Struct. Biol..

[13] Goodsell D.S. (2010). The Machinery of Life.

[14] Falk M., Krone M., Ertl T. (2013). Atomistic visualization of mesoscopic whole-cell simulations using ray-casted instancing. Comput. Graph. Forum.

[15] Parulek J., Jönsson D., Ropinski T., Bruckner S., Ynnerman A., Viola I. (2014). Continuous levels-of-detail and visual abstraction for seamless molecular visualization. Comput. Graph. Forum.

[16] Saito M., Takahashi T. (1990). Comprehensible rendering of 3-d shapes. ACM Siggraph Comput. Graph..

[17] Luft T., Colditz C., Deussen O., Takahashi T. (2006). Image enhancement by unsharp masking the depth buffer. ACM TOG.

[18] Tarini M., Cignoni P., Montani C. (2006). Ambient occlusion and edge cueing for enhancing real time molecular visualization. IEEE Trans. Vis. Comput. Graph..

[19] Krone M., Stone J.E., Ertl T., Schulten K. (2012). Fast visualization of gaussian density surfaces for molecular dynamics and particle system trajectories. EuroVis-Short Papers 2012.

[20] Sharma A., Kalia R., Nakano A., Vashishta P., Montani C. (2004). Scalable and portable visualization of large atomistic datasets. Comput. Phys. Commun..

[21] Grottel S., Reina G., Dachsbacher C., Ertl T. (2010). Coherent culling and shading for large molecular dynamics visualization. Comput. Graph. Forum.

[22] Lampe O.D., Viola I., Reuter N., Hauser H. (2007). Two-level approach to efficient visualization of protein dynamics. IEEE Trans. Vis. Comput. Graph..

[23] Muzic M.L., Viola I. (2014). Illustrative visualization of molecular reactions using omniscient intelligence and passive agents. Comput. Graph. Forum.

[24] Lee J., Park S., Kim J.I. View-dependent rendering of large-scale molecular models using level of detail. Proceedings of the International Conference on Hybrid Information Technology.

[25] Kanai T., Ohtake Y., Kase K. (2006). Hierarchical error-driven approximation of implicit surfaces from polygonal meshes. Eurographics Symposium on Geometry Processing.

[26] Lindow N., Baum D., Hege H.C., Hauser H. (2012). Interactive rendering of materials and biological structu es on atomic and nanoscopic scale. Comput. Graph. Forum.

[27] Grottel S., Krone M., Müller C., Reina G., Ertl T. (2015). Megamol: A prototyping framework for particle-based visualization. IEEE Trans. Vis. Comput. Graph..

[28] Krone M., Bidmon K., Ertl T. (2009). Interactive visualization of molecular surface dynamics. IEEE Trans. Vis. Comput. Graph..

[29] Gralka P., Becher M., Braun M. (2019). MegaMol—A comprehensive prototyping framework for visualizations. Eur. Phys. J. Spec. Top..

[30] Guo D.L., Nie J.L., Liang M., Wang Y., Wang Y.F., Hu Z.P. (2015). View-dependent level-of-detail abstraction for interactive atomistic visualization of biological structures. Comput. Graph..

[31] Klein T., Ertl T. Illustrating Magnetic Field Lines using a Discrete Particle Model. Proceedings of the Vision, Modeling, and Visualization Conference 2004 (VMV 2004).

[32] Kumar P., Yildirim E.A. (2005). Minimum-Volume Enclosing Ellipsoids and Core Sets. J. Optim. Theory Appl..

[33] Gumhold S., Ertl T. Splatting illuminated ellipsoids with depth correction. Proceedings of the Vision, Modeling, and Visualization Conference 2003 (VMV 2003).

[34] Abrahamsson E., Plotkin S.S. (2009). Biovec: A program for biomolecule visualization with ellipsoidal coarse-graining. J. Mol. Graph. Model..

